# Light-driven continuous rotating Möbius strip actuators

**DOI:** 10.1038/s41467-021-22644-9

**Published:** 2021-04-20

**Authors:** Zhen-Zhou Nie, Bo Zuo, Meng Wang, Shuai Huang, Xu-Man Chen, Zhi-Yang Liu, Hong Yang

**Affiliations:** 1 Jiangsu Province Hi-Tech Key Laboratory for Bio-medical Research, State Key Laboratory of Bioelectronics, School of Chemistry and Chemical Engineering, Southeast University, Nanjing, China; 2 Institute of Advanced Materials, Southeast University, Nanjing, China

**Keywords:** Actuators, Liquid crystals

## Abstract

Twisted toroidal ribbons such as the one-sided Möbius strip have inspired scientists, engineers and artists for many centuries. A physical Möbius strip exhibits interesting mechanical properties deriving from a tendency to redistribute the torsional strain away from the twist region. This leads to the interesting possibility of building topological actuators with continuous deformations. Here we report on a series of corresponding bi-layered stripe actuators using a photothermally responsive liquid crystal elastomer as the fundamental polymeric material. Employing a special procedure, even Möbius strips with an odd number of twists can be fabricated exhibiting a seamless homeotropic and homogeneous morphology. Imposing a suitable contraction gradient under near-infrared light irradiation, these ribbons can realize continuous anticlockwise/clockwise in-situ rotation. Our work could pave the way for developing actuators and shape morphing materials that need not rely on switching between distinct states.

## Abstract (web summary)

Shape morphing materials are usually difficult to operate in a continuous fashion. Nie et al. fabricate stripes with liquid crystalline elastomers that can be given a Möbius-like morphology with seamless material composition, and perpetually driven under photothermally induced actuation.

## Introduction

Möbius strip^1,2^, a two-dimensional circular structure obtained by twisting a ribbon and then joining the two ends together, is undoubtedly one of the most fascinating objects that has inspired mathematicians, physicians, chemists, engineers and artists for many centuries^3–9^. As August Ferdinand Möbius noted in his original manuscript^1^, circular bands with an odd number of twists (*T*_w_, defined as the number of 180° rotations)^10^ exhibit one side and one edge, whereas other toroidal ribbons with an even number of twists possess two sides and two edges^11^. Strictly speaking, only the former single-faced twisted toroidal ribbons are regarded as Möbius strips.

All Möbius strips have a C2 symmetry axis, on which there is a locus of twist point where the edges pass through the axial plane. Due to the twisted toroidal conformation, Möbius ribbons have considerable torsional strains, and the highest bending/torsional energy is distributed around the locus of the twist region^12^. Trapping such twist strain within a topological structure is beneficial for enhancing the actuation performance of the corresponding actuator^13^. These interesting scientific discoveries inspire us: if a Möbius strip was prepared from stimulus-responsive polymeric materials^13–23^, the locus of twist region would become the strongest actuation responsive point, and then, applying stimuli on the locus of the twist region might spread the torsional energy along with the twisted band, and further induce a continuous deformation, i.e., rotation of twist along with the ribbon.

Recently, Ziebert, Kulić and co-workers realized a continuous rotary motion in mechanically prestrained elastic torus polymer rods^24^. In their wheel within the material model, two factors, topological prestrain and rotational-symmetry breaking (i.e., compression/tension in the inner/outer sides of the torus rod^24^), would give rise to a zero-elastic energy mode (ZEEM), were essential to the continuum material deformation. This ZEEM theory could explain the outcome of a recent Möbius strip actuator example demonstrated by Lewis and colleagues, which showed simple shrinkage of the strip, instead of continuous rotation, under thermal stimulus^25^ because the inside and outside faces of the Möbius strip actuator were identical, which could not generate symmetry breaking to further induce a continuous rotary motion.

Here, we show a series of light-guided continuous rotating Möbius strip actuators based on a one-sided, defect-free, homeotropic and homogeneous photothermal-contraction-gradient structure design, which induces symmetry breaking in both the homeotropic and homogeneous directions of the twisted strip. These Möbius strip actuators can realize continuous anticlockwise/clockwise in situ rotation under near-infrared (NIR) light irradiation.

## Results and discussion

### Definition and objective

**Fig. 1:**
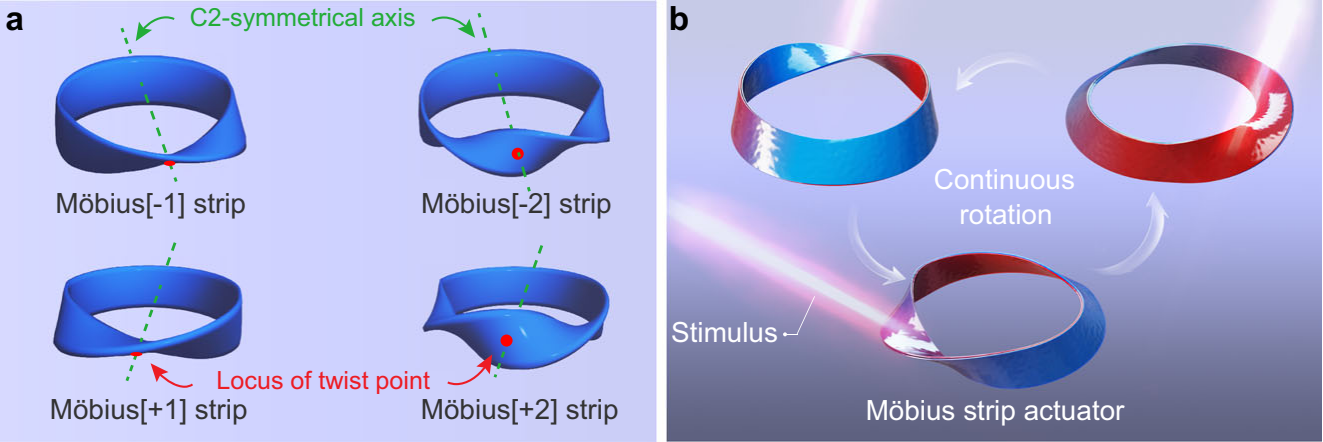
A stimulus-guided continuous rotating Möbius strip actuator. **a** Schematic illustration of the C2 symmetry axis and locus of twist point of Möbius[±1] and Möbius[±2] strip models. Möbius[±*T*_w_] strip defines all the Möbius and twisted toroidal ribbons. *T*_w_ is defined as the number of 180° rotations, *T*_w_ = 1 or 2; the positive or negative sign corresponds to a left-handed or right-handed helix. **b** Diagrammatic drawing of the desired in situ continuous rotation scenario of the Möbius strip actuator under an external stimulus. The colour gradient from red to blue represents the contraction capability gradient (the red and blue parts indicate high and low contraction capabilities, respectively).

Here, to simplify the nomenclature, we use Möbius[±*T*_w_] to define all the Möbius and twisted toroidal ribbons reported in this manuscript. The positive or negative sign corresponds to a left-handed or right-handed helix. The C2 symmetry axis and the locus of twist point of the Möbius[±*T*_w_] strips are schematically illustrated in Fig. 1a. To achieve a stimulus-guided continuous in situ rotation of the corresponding Möbius strip actuator is the objective of this work, as presented in Fig. 1b.

### Stimulus-responsive behaviour of single-layered Möbius strip actuators

To prepare the desired Möbius strip actuators, we chose a liquid crystal elastomer (LCE) as the fundamental stimulus-responsive polymeric material because LCEs, as classical and predominant two-way shape-memory materials, can exhibit reversible, complex and huge amplitude shape deformations along with the external-stimuli-triggered order–disorder phase transition of the internal mesogenic polymer network and have prosperous application prospects in smart actuators, robotic technology and biomedical engineering^23,26–37^. Moreover, we embedded a NIR absorbing dye into the LCE matrix so that the prepared ribbon actuators could be stimulus triggered by remote light control, relying on the photothermal conversion effect of the incorporated NIR dye^38^.

**Fig. 2:**
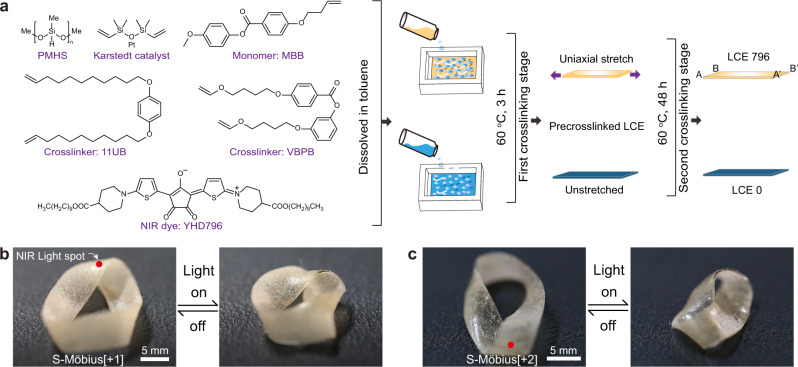
Chemical composition and actuation behaviour of single-layered actuators. **a** Schematic illustration of the chemical components of this LCE system and the preparation procedures of LCE796 (orange part) and LCE0 (dark blue part) ribbons. Photographs of the single-layered **b** S-Möbius[+1] and **c** S-Möbius[+2] actuators upon application of a photothermal stimulus (scale bars: 5 mm). Supplementary Movie 1 shows the scenario in motion.

The chemical composition of the LCE material is presented in Fig. 2a. Poly(methylhydrosiloxane) (PMHS) and 4-methoxyphenyl-4-(1-buteneoxy)benzoate (MBB) were treated as the macromolecular backbone and mesogenic monomer, respectively. Two crosslinkers, 4-bis-undec-10-enyloxy-benzene (11UB) and 4-[4-(vinyloxy)butoxy]phenyl 4-[4-(vinyloxy)-butoxy]benzoate (VBPB), with varied molar ratio could be used to tune the LC-to-isotropic phase transition temperature (*T*_iso_) of the corresponding LCE ribbon. The croconaine dye YHD796 with a maximum NIR absorption at 796 nm fulfilled the photothermal conversion duty, and Karstedt’s catalyst (platinum(0)-1,3-divinyl-1,1,3,3-tetramethyldisiloxane complex solution in xylene, Pt ~2%) was used to trigger the hydrosilylation reaction. The detailed synthetic protocol is described in the Supplementary Information. Here, we prepared two kinds of LCE ribbons: LCE796 and LCE0. The LCE796 ribbon was the YHD796 dye-embedded, monodomain LCE material that was uniaxially stretched during the two-step crosslinking process^39^, whereas the polydomain LCE0 ribbon underwent neither mechanical programming treatment nor photothermal dye incorporation.

With the polysiloxane LCE material, we first fabricated two single-layered Möbius strip actuators (S-Möbius[+1] and S-Möbius[+2]) by clockwise twisting the AB end of the LCE796 ribbon either 180° or 360° and then gluing it in a head-to-head manner to the other A’B’ end by using a silicone adhesive (HJ-T326, Dongguan Fangguan Industrial Materials Co. Ltd.) to form a toroidal ring. When NIR light (centre wavelength: 808 ± 3 nm, output power: 8 W) was irradiated on the locus of twist regions of S-Möbius[+1] and S-Möbius[+2] ribbons, the two ribbons shrunk and wrinkled, as shown in Fig. 2b, c and Supplementary Movie 1, while the desired rotation scenario was not observed, consistent with the literature results^25^. Directly heating the S-Möbius[+1] and S-Möbius[+2] ribbons on a hot stage would induce the same phenomenon, possibly derived from the uniform in-plane shrinkage of the single-layered LCE796 ribbon, which could not change the curvature of the locus of twist region or further break the energy localization equilibrium to induce a continuous rotation of twist along the Möbius strips.

### Stimulus-responsive behaviour of bilayered Möbius strip actuators

**Fig. 3:**
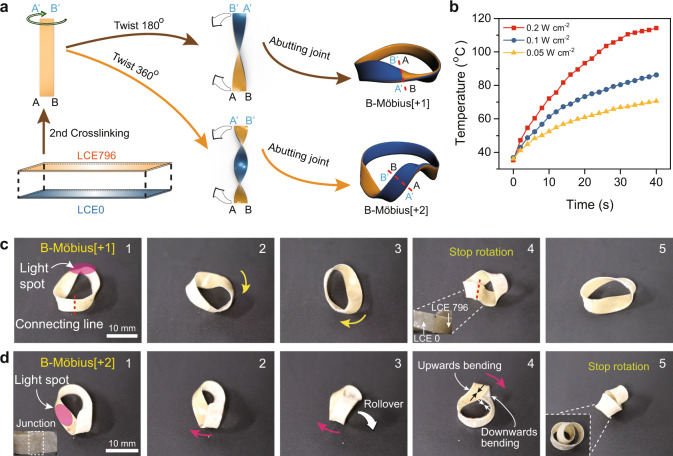
Preparation procedure and actuation behaviour of bilayered actuators. **a** Schematic illustration of the fabrication procedures of B-Möbius[+1] and B-Möbius[+2] ribbon actuators. **b** Real-time surface temperature profiles of the bilayered LCE film stimulated by NIR light of various intensities. Photographs showing the shape deformations of **c** B-Möbius[+1] and **d** B-Möbius[+2] ribbon actuators under the clockwise stimulation of NIR light irradiated on their locus of twist regions (scale bars: 10 mm). Supplementary Movie 2 shows the scenario in motion.

Learning from this single-layered actuator trial, we turned our attention to bilayered Möbius strip actuators, the inside and outside layers of which would contract differently under a photothermal stimulus, causing the bending curvature of the light-exposed region to change so that the stress balance of the twisted ribbon would be disrupted, the strain energy localization would be redistributed, and the desired rotation of twist along the ribbon might be achieved. Following this logic, we synthesized three bilayered Möbius strip actuators (B-Möbius[+1], B-Möbius[+2] and B-Möbius[−2]). The fabrication method is schematically illustrated in Fig. 3a. The bilayered LCE ribbon was prepared by placing a pre-crosslinked, uniaxially stretched LCE796 strip on top of a pre-crosslinked LCE0 film (Supplementary Fig. 1), followed by heating in an oven at 60 °C for 2 days, which would spontaneously adhere the LCE796 and LCE0 layers together during the second-step hydrosilylation crosslinking procedure. Subsequently, one end of the straight bilayered LCE strip (38.0 mm long and 4.0 mm wide) was twisted clockwise either 180° or 360° and further glued in a head-to-head manner to the other end by using a silicone adhesive to provide either a B-Möbius[+1] or a B-Möbius[+2] ribbon actuator. Similarly, an anticlockwise twist of the LCE strip by 360° yielded a B-Möbius[-2] ribbon actuator instead. It is worth noting that the resulting B-Möbius[+1] ribbon with two sides and one edge was not a Möbius strip in the true sense but a Möbius-shaped strip, whereas the prepared B-Möbius[+2] sample fully complied with the twisted toroidal ribbon definition^1,11^.

Benefiting from the photothermal conversion effect of the incorporated NIR dye YHD796 (0.07 wt%), the fundamental bilayered LCE film could be efficiently heated above the *T*_iso_ temperature of LCE796 (69.5 °C, Supplementary Fig. 2) within 10 s when the NIR light intensity reached 0.2 W cm^−2^, as demonstrated in Fig. 3b. Beyond the LC-to-isotropic phase transition, the monodomain LCE796 film drastically contracted, whereas the shape of LCE0 remained almost the same. This homeotropic contraction ratio divergence caused the bilayered LCE material to bend towards the LCE796 layer and consequently changed the curvature of the locus of twist region of the corresponding toroidal ribbons.

To investigate the light-triggered shape morphing behaviour of these toroidal ribbon actuators, we placed B-Möbius[+1] and B-Möbius[+2] ribbons horizontally on a table and applied 808 nm NIR light to irradiate the locus of twist regions of these two ribbons. As shown in Fig. 3c, d and Supplementary Movie 2, rotation of B-Möbius[+1] and B-Möbius[+2] ribbon actuators was observed when 808 nm NIR light stimulated the locus of twist regions and was moved clockwise along the circular rings. However, the clockwise rotation of the B-Möbius[+1] ribbon actuator stopped when the locus of twist point reached the end-to-end junction where the inside and outside layers were no longer successive, which could be regarded as a defect point, as depicted in Fig. 3c.

Under NIR illumination, the locus of twist of the B-Möbius[+2] ribbon rotated along with the circular band until a critical point where the twisted ribbon transformed into a helically writhed loop, as demonstrated in Fig. 3d and Supplementary Movie 2. In detail, at the starting point, the inside and outside layers in the locus of twist region of the B-Möbius[+2] ribbon were LCE796 and LCE0, respectively, so the twist region should bend towards the inner layer and be forced to rotate clockwise under a photothermal stimulus. After the locus of twist, region was rotated by ca. 180° around the circular band, the inside and outside layers of the twist region inversely changed to LCE0 and LCE796 so that the twist region bent towards the outer layer, which opened the twist and released the torsional strain energy by winding the ribbon helically around itself to form a more stable writhed loop, which eventually stopped the rotation.

This phenomenon actually complied with the theory that higher-order Möbius strips were elastically unstable and featured self-contacted writhed loop structures^40^. In general, B-Möbius[2] ribbons can be reshaped into two different helically writhed loops: a heart-shaped loop and a figure-eight loop (Supplementary Fig. 3), depending on the ribbon length/width ratio. A length/width ratio of ca. 11 was the boundary at which longer and narrower B-Möbius[2] ribbons would morph into figure-eight writhed loops, while shorter and broader Möbius[2] ribbons would change into heart-shaped writhed loops (Supplementary Discussion).

### Continuous rotating B-Möbius[±2] strip actuators

**Fig. 4:**
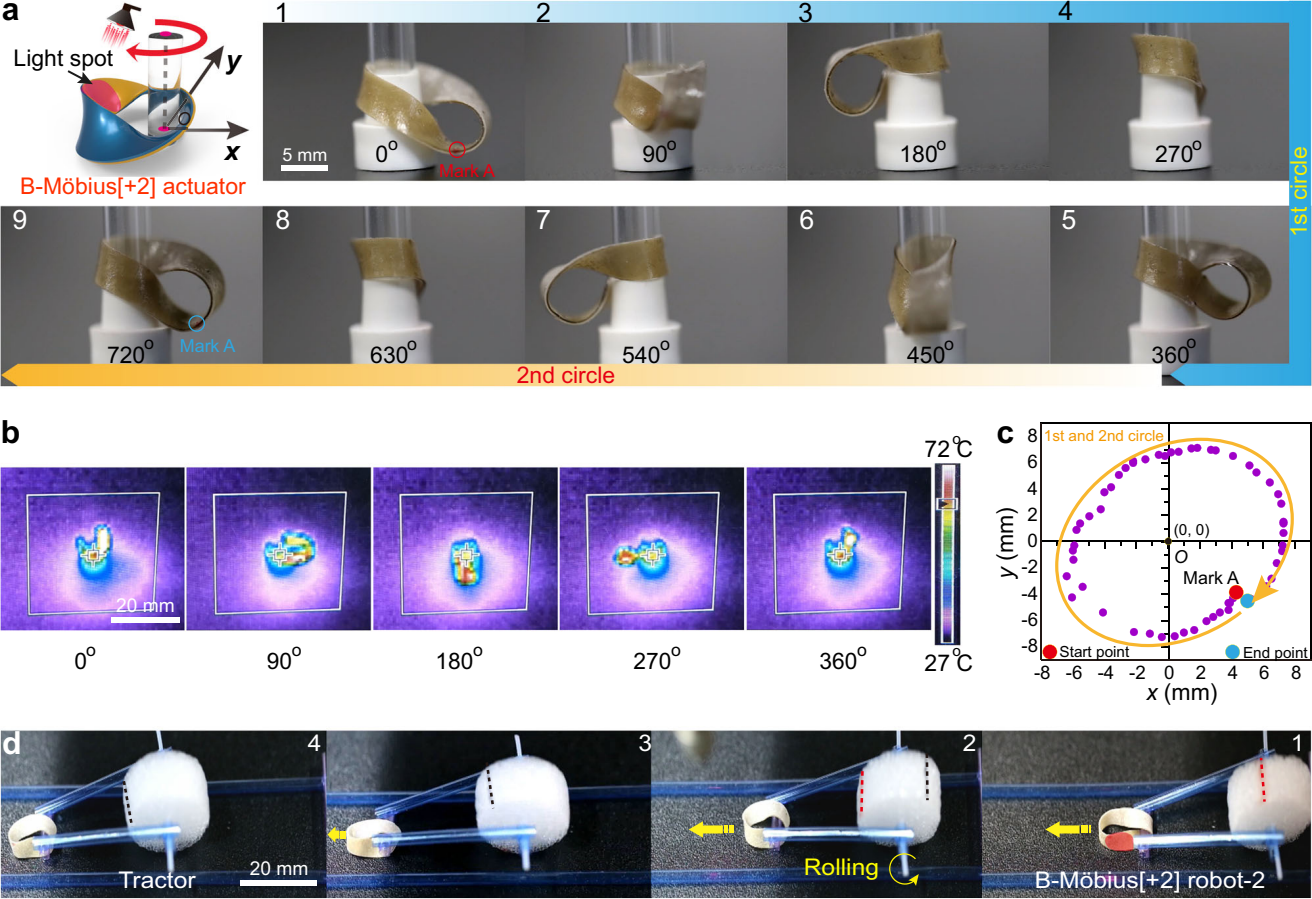
NIR light-guided clockwise rotation of the B-Möbius[+2] actuator. **a** Photographs of continuous clockwise rotation of one B-Möbius[+2] actuator around a circular cylinder under irradiation by NIR light (scale bar: 5 mm). Supplementary Movie 3 shows the scenario in motion. **b** Real-time temperature distribution in the B-Möbius[+2] ribbon during clockwise rotation. The colour bar represents the real-time object temperature (scale bar: 20 mm). **c** Position coordinates of the point denoted mark A on the B-Möbius[+2] ribbon recorded during a continuous 720° clockwise rotation. **d** Photographs of a light-fuelled B-Möbius[+2] tractor dragging a foamy cylinder (scale bar: 20 mm). Supplementary Movie 9 shows the scenario in motion.

To induce a continuous rotation of twist motion of these B-Möbius[±2] ribbon actuators, we placed a circular cylinder inside the toroidal strips to sterically hinder the transformation from double-twisted bands to helically writhed loops. As illustrated in Fig. 4a, a diameter-matched circular cylinder (*d* = 5.0 mm) was placed inside one B-Möbius[+2] strip (length: 38.0 mm, width: 4.0 mm). When the locus of twist region of the B-Möbius[+2] actuator was irradiated by 808 nm NIR light, the temperature of the twist region quickly jumped above the *T*_iso_ temperature of LCE796 (Fig. 4b), causing a variation in the bending curvature of the exposed ribbon region, which forced the twist region to move away from the original location. With the laser spot circuiting clockwise, the B-Möbius[+2] actuator continuously rotated around the inner circular cylinder at a rotation speed of ca. 16° s^−1^, as shown in Supplementary Movie 3.

To investigate the rotation motion statistically, the real-time position coordinates of one point, mark A, under 808 nm light stimulation were analysed by Tracker software and are plotted in Fig. 4c. We set the vertical projection of the midpoint of the circular cylinder as the origin (0, 0) in the *x-y* plane and recorded the position coordinates of the vertical projection of mark A during the clockwise rotation. When mark A underwent one full elliptical circuit and returned to the original position, the B-Möbius[+2] actuator rotated approximately ca. 720° around the central cylinder clockwise, which could be more clearly visualized in a view from the overlook, as shown in Supplementary Fig. 4 and Supplementary Movie 4. In addition, the actuation of the B-Möbius[−2] ribbon actuator that could perform anticlockwise rotation at a speed of ca. 11° s^−1^ was also examined, as shown in Supplementary Fig. 5 and Supplementary Movie 5.

Encouraged by the above results, we fabricated two types of light-fuelled rolling robots (i.e., B-Möbius[±2] robot-1 and B-Möbius[±2] robot-2) by placing a hollow plastic tube in one of the two Möbius[±2] holes with the inner layer composed of either LCE0 or LCE796, respectively, as schematically illustrated in Supplementary Fig. 6a. The locomotion modes of the two Möbius[±2] robots were relevant to the position of the cylindrical tube placed in the ribbon and the targeted light-trigger region (Supplementary Discussion). Basically, B-Möbius[+2] robot-1 could realize either clockwise or anticlockwise rotation when either the outside or inside ribbon region closely wrapped around the cylindrical tube was exposed to NIR light (Supplementary Fig. 6b–d and Supplementary Movies 6, 7). B-Möbius[±2] robot-2 could move along a curvilinear trajectory because of the anticlockwise/clockwise rotation sliding of the B-Möbius[±2] strip under NIR light stimulation of a position near the twist region of the B-Möbius[±2] actuator (Supplementary Fig. 7 and Supplementary Movie 8).

Furthermore, inspired by the Ikeda group’s light-driven plastic motor based on a cylindrical LCE pulley system^26^, we applied B-Möbius[+2] robot-2 as a light-fuelled tractor (weight: 70 mg) that could drag a foamy cylinder (weight: 183 mg) and roll forward inside a defined orbit at a speed of 0.51 mm s^−1^, as plotted in Fig. 4d and shown in Supplementary Movie 9.

### Continuous rotating Möbius[±1] strip actuators

**Fig. 5:**
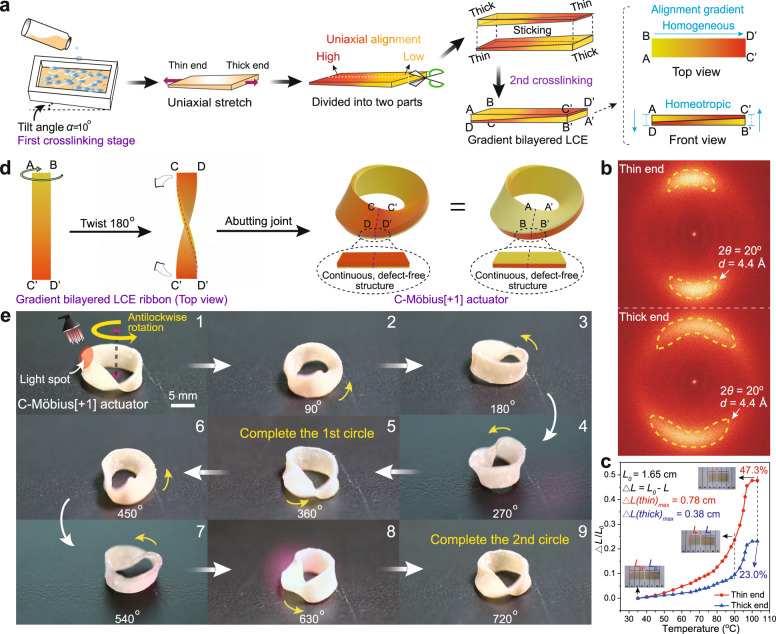
Photoguided in situ continuous rotation of the C-Möbius[+1] actuator. **a** Schematic illustration of the preparation procedure of the bilayered LCE ribbon with a homogeneous and homeotropic mesogen alignment gradient. The colour gradient from red to yellow represents the uniaxial alignment gradient (the red part indicates high uniaxial alignment, and the yellow part indicates low uniaxial alignment, same as in (**d**)). **b** 2D WAXS patterns of the thin and thick ends of the LCE film. **c** Photothermal shrinkage ratios of the thin and thick ends of the LCE film during the heating process. **d** Schematic illustration of the fabrication procedure of the C-Möbius[+1] strip actuator with a homogeneous and homeotropic alignment gradient. **e** Photographs of the continuous anticlockwise rotation of the C-Möbius[+1] strip actuator under irradiation with NIR light (scale bar: 5 mm). Supplementary Movie 10 shows the scenario in motion.

Despite all the above investigations, the most challenging and exhilarating task was to create a continuous rotating B-Möbius[±1] strip actuator with a continuous rotation of twist motion. In the previous B-Möbius[+1] ribbon case, on the one hand, the homeotropic photothermal contraction gradient was necessary for the Möbius strip to drive the rotation of twist; on the other hand, the two different homeotropic photothermal-contractive LCE sides and one edge structure would induce a defect point, where the inside and outside LCE layers were no longer successive, and eventually quench the rotation. To solve this dilemma, we designed an authentic Möbius[±1] strip actuator with a one-sided, defect-free, homeotropic and homogeneous photothermal-contraction-gradient surface, as shown in Fig. 5. Thereafter, we named these continuous-structured Möbius[±1] strip actuators as C-Möbius[±1] strip actuators.

The synthetic protocol is schematically illustrated in Fig. 5a. During the first hydrosilylation crosslinking stage, a PTFE rectangular mould was placed on an inclined surface with a tilt angle of 10° so that the resulting pre-crosslinked LCE film would have a continuous thickness gradient along the longitudinal direction. The pre-crosslinked LCE film was then uniaxially stretched to ca. 140% of its original length, and the colour gradient of the ribbon was used to represent the mesogen alignment gradient of the LCE film. Subsequently, the LCE sample was sliced along the longitudinal direction into two symmetric pieces, which were then overlapped with the thin and thick ends of the top piece opposingly adhered onto the thick and thin ends of the bottom piece and further subjected to the second-step crosslinking stage to provide a gradient bilayered LCE ribbon (33.0 mm long and 3.0 mm wide). As demonstrated in Supplementary Fig. 8, the thicknesses of the thin and thick ends of the LCE film were ca. 102–109 μm and 141–147 μm.

The logic of this treatment is that under the same external force, the thin end of the LCE ribbon would be stretched longer and have a higher chain anisotropy than the thick end so that the LCE ribbon would have a homogeneous mesogen alignment gradient and a corresponding homogeneous photothermal contraction gradient. As illustrated in Fig. 5b and Supplementary Fig. 9, the molecular orientations of the thin and thick ends of the LCE film were characterized by utilizing two-dimensional wide-angle X-ray scattering (2D-WAXS). The 2D-WAXS patterns recorded at 30 °C with the incident beam perpendicular to the film surface showed a much narrower crescent of the thin end at 2*θ* = 20° compared to that of the thick end. Furthermore, the orientational order parameters (*S*)^41^ of the thin and thick ends of the LCE film were calculated as 0.70 and 0.55, respectively, which implied a significant mesogen alignment gradient along the long axis direction of the film sample.

The photothermal contraction gradient of the LCE sample was also demonstrated by measuring the photothermal shrinkage ratio (∆*L*/*L*_*0*_) of the thin and thick ends of the LCE ribbon with increasing temperature, where *L*_*0*_ was the original length (ca. 1.65 cm) between either the thin or thick LCE end and the middle point of the ribbon at 35 °C, *L* was the varied length between either the thin or thick LCE end and the original middle point of the ribbon at any specific temperature, and ∆*L* = *L*_*0*_ − *L* was set as the contracted length of each LCE end (Fig. 5c). The shrinkage ratio difference between the two LCE ends increased with increasing temperature and reached the maximal value of 24.3% (thin end: 47.3%, thick end: 23.0%) beyond 100 °C, which provided solid proof for the existence of a homogeneous photothermal contraction gradient between the thin and thick ends of the LCE ribbon.

The gradient bilayered LCE ribbon, as shown in Fig. 5d, was clockwise twisted by 180° and then glued in a head-to-head manner. In this abutting joint, the thin LCE end was connected with the other thin LCE end, while the thick LCE part was glued with the other thick LCE part so that the prepared Möbius[+1] strip had a continuous, one-sided, defect-free, homeotropic and homogeneous photothermal-contraction-gradient structure. The radius (*R*) and width (2*w*) of the C-Möbius[+1] strip were ca. 4.8 mm and 3.0 mm, respectively; the *R*/*w* ratio was ca. 3.2 (Supplementary Fig. 10 and Supplementary Table 1).

In this case, the C-Möbius[+1] strip actuator bearing no defect point could achieve the desired continuous, anticlockwise, in situ rotation along with the circular band at a speed of ca. 4.9° s^−1^ under NIR light irradiation, as shown in Fig. 5e and Supplementary Movie 10.

**Fig. 6:**
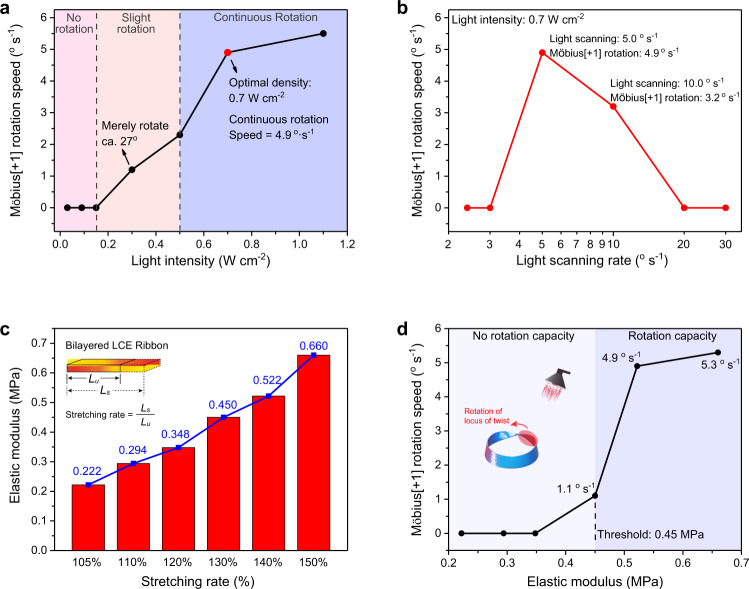
The correlation between NIR light intensity, light scanning rate, ribbon elasticity and the rotation speed of C-Möbius[+1] actuators. **a** Rotation speed of a Möbius actuator as a function of 808 nm NIR light intensity. **b** Rotation speed of a Möbius actuator plotted against the NIR light scanning rate. **c** Elastic modulus of the bilayered LCE ribbon with varied stretching rate. **d** Actuator rotation speed diagram plotted against the varied elastic modulus of bilayered LCE sample.

The rotation speed of the C-Möbius[+1] strip actuator depended mainly on the NIR light intensity, light scanning rate and the ribbon’s elasticity. Figure 6a and Supplementary Fig. 11 describe the correlation between the rotation speed of the C-Möbius[+1] strip actuator and the varied NIR light intensity (ranging from 0.03 to 1.1 W cm^−2^). If NIR light with low optical intensity (0.2–0.5 W cm^−2^) were applied (Supplementary Fig. 11b, c), the Möbius actuator would exhibit a discontinuous rotation with a slow rotation speed (1.2–2.3° s^−1^). When the illumination intensity was increased to 0.7 W cm^−2^, the twisted band achieved a continuous rotation at a speed of 4.9° s^−1^ (Supplementary Fig. 11d). Thermal images indicated that the locus of the twist region was maintained at ~83 °C during the rotation process, which was the critical temperature to form a successive rotation. When the light intensity reached ca. 1.1 W cm^−2^, the rotation speed of the Möbius actuator further increased to 5.5° s^−1^ (Supplementary Fig. 11e). However, since high light intensity would cause serious thermal damage to the LCE ribbons, a moderate light intensity of 0.7 W cm^−2^ was set as the optimal power for driving the continuous rotation of the Möbius strip actuators.

As presented in Fig. 6b, the light scanning rate governed the local photothermal response and markedly influenced the rotation speed of the C-Möbius[+1] actuator. When the NIR light intensity was set as 0.7 W cm^−2^, the scanning rate was tuned to 2.4, 3.0, 5.0, 10, 20, and 30° s^−1^, while the surface temperature of the locus of twist region reached ca. 140 °C, 105 °C, 83 °C, 70 °C, 60 °C, and 45 °C, respectively (Supplementary Fig. 12). Beyond 105 °C, the actuator became very soft and had no deformation capability (Supplementary Fig. 12a, b). At the critical temperature of around 83 °C, adequate photothermal conversion could drive the continuous rotation of the Möbius actuator at a speed of 4.9° s^−1^ (Supplementary Fig. 11d). Below 70 °C, the short time scale of light irradiation on the locus of twist caused the discontinuous deformation of the actuator with a slow rotation speed (0–3.2° s^−1^) (Supplementary Fig. 12c–e).

To investigate the influence of the elasticity property of the LCE ribbon on the rotation speed of the Möbius actuator, six bilayered LCE samples with varied stretching rate were prepared and their elastic moduli were examined by using a dynamic mechanical analyzer (DMA850, TA Instrument) with a tension clamp for the quasi-static stress-strain measurement at 25 °C (Supplementary Fig. 13). As illustrated in Fig. 6c, the stretching rate, defined as *L*_*s*_/*L*_*u*_ (*L*_*u*_ was the original un-stretched ribbon length and *L*_*s*_ was the stretched ribbon length) along the longitudinal direction, ranged from 105 to 150%. The corresponding elastic moduli of the bilayered polysiloxane LCE ribbons increased from 0.222 to 0.660 MPa in a quasi-linear manner.

As demonstrated in Fig. 6d, the C-Möbius[+1] actuators with elastic modulus range of 0.22–0.35 MPa were not capable of performing a continuous rotation. When the elastic modulus reached a threshold value of 0.45 MPa, the Möbius actuator could start to rotate at a slow speed of 1.1° s^−1^ (Supplementary Fig. 13d). When the elastic modulus was further raised to 0.52 and 0.66 MPa, the rotation speed increased to 4.9 and 5.3° s^−1^, respectively. As a result, the rotation capacity of the Möbius strip actuator was positively correlated with the stretching ratio and the elastic modulus (within a range from 0.45 to 0.66 MPa) of the bilayered polysiloxane LCE samples. It is worth noting here that a high stretching rate of ca. 150% sometimes might cause breakage of the LCE ribbons. Therefore, we chose 140% as the optimal stretching rate for the bilayered LCE ribbons to be used to fabricate the Möbius strip actuators.

**Fig. 7:**
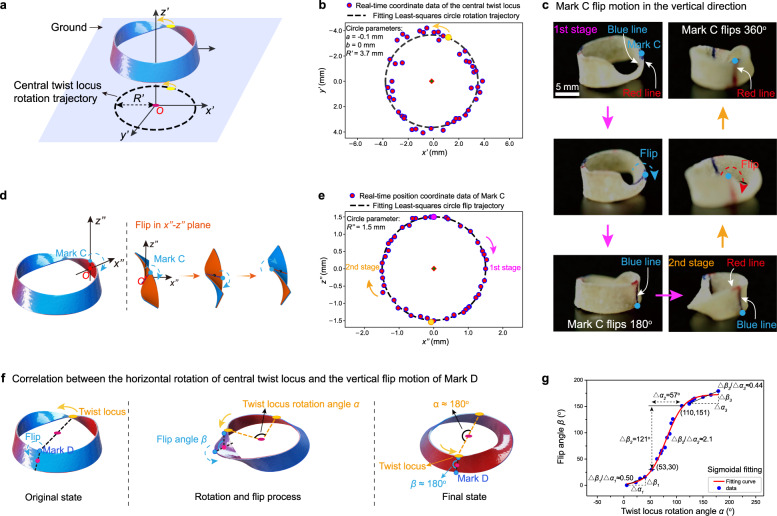
Statistical analysis of the motion dynamics of the C-Möbius[+1] actuator. **a** Schematic illustration of the rotation of the locus of twist point projected in the *x’-y’* coordinates. The colour gradient from red to blue represents the contraction capability gradient (the red and blue parts indicate high and low contraction capabilities, respectively, same as in (**d**, **f**)). **b** Rotation trajectory of the locus of twist point of the C-Möbius[+1] actuator (all scatter spot data were recorded every 3 s). **c** Photographs of the flip of the blue/red lines normal to the longitudinal direction of the strip (scale bar: 5 mm). Supplementary Movie 11 shows the scenario in motion. **d** Diagrammatic drawing of the flip motion of the C-Möbius[+1] actuator in the *x”-z”* plane. **e** The position coordinates of the point denoted mark C of the C-Möbius[+1] ribbon actuator recorded along a circular trajectory (all scatter spot data were recorded every 2 s). **f** Schematic illustration of the correlation between the rotation of twist (yellow point) and the turning motion of the point mark D (blue point). **g** Flip angle *β* as a function of the twist locus rotation angle *α* and Boltzmann model fit.

To further understand the motion dynamics of the C-Möbius[+1] actuator, the motion paths of the points at the rim of the C-Möbius[+1] band were tracked and analysed. First, the locus of twist point (yellow point) of the C-Möbius[+1] actuator was projected in the horizontal *x*′-*y*′ plane, as illustrated in Fig. 7a. The central twist point would return to the original position after 720° anticlockwise rotation. The real-time coordinate data of the central twist point were recorded for fitting the circle by using the least-squares method. Figure 7b indicated that the locomotion of the central twist point nearly followed a circular trajectory, which is presented in Eq. (1)

1 $${(x^{\prime} -a)}^{2}+{(y^{\prime} -b)}^{2}={R^{\prime2}}$$

where the radius *R’* is 3.7 mm, and parameters *a* and *b* are −0.1 mm and 0 mm, respectively. It is worth noting that the photothermal conversion effect induced a longitudinal contraction of the soft LCE ribbon, and subsequently resulted in the significant decrease of the radius of the motion trace in comparison with that (4.8 mm) of the original C-Möbius[+1] actuator.

Second, any specific point on the Möbius strip executed a nearly in situ flip motion in the vertical direction. The inner and outer surfaces of the C-Möbius[+1] ribbon were marked with a blue line and a red line normal to the long axis of the strip, respectively, and one point, mark C, was located at the junction of the blue line and red line. Figure 7c shows that in the first circle rotation, the inner and outer lines changed to red and blue lines, and mark C rotated 180° During the second circle rotation, the outer blue line rotated to the inner surface, and mark C returned to the original position after completing a 360° rotation. To dissect the rotation statistically, the position coordinates of mark C relative to midpoint O of the width of the Möbius strip were analysed and are plotted in Fig. 7d. We set the midpoint O of the width as the origin (0, 0) in the vertical *x*′′-*z*′′ plane and recorded the relative position coordinates of mark C during the rotation, which confirmed that the point on the ring edge was capable of moving along a roughly circular trajectory in the *x*′′-*z*′′ plane (Fig. 7e and Supplementary Movie 11). Furthermore, when mark C completed a 360° rotation, the locus of twist would have undergone two 360° rotation circuits. The flip circle trace was obtained by using Eq. (2) based on the least-squares method, and the trace radius *R”* was calculated as 1.5 mm.

2 $${x^{\prime\prime2}}+{z^{\prime\prime 2}}={R^{\prime\prime2}}$$

Third, the correlation between the horizontal rotation of the locus of twist point and the vertical flip motion of a specific point on the twisted band was investigated. As shown in Fig. 7f, the locus of twist point is marked in yellow, mark D is a point on the edge of the twisted band passing through the C2 symmetry axis plane, angle *α* is defined as any specific rotation angle of the locus of twist point in the horizontal plane, and angle *β* is defined as the flip angle of mark D in the vertical plane. As the anticlockwise twist locus rotation angle *α* increased, mark D turned outward and the corresponding flip angle *β* increased as well. When the locus of twist point rotated 180° to reach the cross-sectional plane containing mark D, mark D realized a half-circle trajectory. For the quantitative analysis of the correlation between the rotation of the twist locus and the turning motion of mark D, the flip angle *β* was plotted against the twist rotation angle *α*. As illustrated in Fig. 7g, the S-shaped growth trend was observed from the scatter data diagram and could be divided into three stages. In the first stage $\alpha \in (0,31)$, $\varDelta \beta /\varDelta \alpha \approx 0.50$ indicated that the locus of twist rotated 2° and at the same time the flip angle *β* could change by ca. 1°, implying a small turning extent. In the second interval $\alpha \in (53,110)$, the flip angle *β* changed from 30° to 151°, and $\varDelta \beta /\varDelta \alpha$ was increased to ca. 2.1. In contrast to the result of the first stage, the flip motion exhibited a faster response to the twist rotation and possessed a larger angle growth rate in the second stage. In the third stage $\alpha \in (124,180)$, the flip motion of mark D was nearly completed, and the angle growth rate decreased to $\varDelta \beta /\varDelta \alpha \approx 0.44$. Moreover, the Boltzmann function was used for fitting the data to obtain a sigmoidal curve (Supplementary Discussion). The curve showed good agreement with the experimental result.

Moreover, miniaturization of the C-Möbius[+1] strip actuator (22.0 mm long and 2.0 mm wide) was realized and the actuator could continuously rotate at a speed of 5.1° s^−1^, which was beneficial to the increase of rotation speed and energy efficiency (Supplementary Fig. 14). In addition, the light-fuelled actuation of the C-Möbius[−1] strip actuator could perform a successive clockwise rotation at a speed of ca. 5.6° s^−1^ was also successfully examined, as shown in Supplementary Fig. 15 and Supplementary Movie 12.

In conclusion, a series of LCE-based Möbius strip actuators capable of executing continuous rotation and rolling motions in response to NIR light stimulation are described in this manuscript. These continuous rotation behaviours of the twisted ribbon actuators are realized by combining the photothermal effect, gradient stress, twisted toroidal geometry and torsional strain of Möbius strips. Moreover, two types of NIR light-fuelled B-Möbius[±2] rolling robots that can rotate clockwise/anticlockwise and move along a curvilinear trajectory are presented by modulating the location of the tube placed inside them and the light-target region.

Most significantly, two authentic single-faced Möbius[±1] strip actuators based on a continuous, defect-free, homeotropic and homogeneous photothermal-contraction-gradient structure were able to realize continuous in situ rotation under NIR irradiation. We hope that this work will pave the way for developing actuators and shape morphing materials that need not rely on switching between distinct states.

## Methods

### General considerations

All the starting materials, instrumentation descriptions, preparation procedures of Möbius strip actuators are shown in Supplementary Methods. All the rotation speeds of Möbius strip actuators are presented in Supplementary Table 2. Supplementary Movie 1 presents the actuation behaviour of S-Möbius[+1] and S-Möbius[+2] strips. Supplementary Movie 2 shows the actuation behaviour of B-Möbius[+1] and B-Möbius[+2] strips under the stimulation of NIR light. Supplementary Movie 3 shows the clockwise rotation of B-Möbius[+2] actuator around a circular cylinder. Supplementary Movie 4 presents a top view of the clockwise rotation of B-Möbius[+2] actuator around a circular cylinder. Supplementary Movie 5 shows the anticlockwise rotation of B-Möbius[−2] actuator around a circular cylinder. Supplementary Movie 6 demonstrates the clockwise and anticlockwise rotation of B-Möbius[+2] robot-1 loaded with a cylindrical tube. Supplementary Movie 7 presents the anticlockwise and clockwise rotation of B-Möbius[−2] robot-1 loaded with a cylindrical tube. Supplementary Movie 8 shows the rolling motions of B-Möbius[+2] robot-2 and B-Möbius[−2] robot-2 driven by NIR light. Supplementary Movie 9 shows a light-fuelled B-Möbius[+2] tractor dragging a foamy cylinder. Supplementary Movie 10 shows the anticlockwise rotation of C-Möbius[+1] actuator. Supplementary Movie 11 indicates a side view of the anticlockwise flip motion of C-Möbius[+1] actuator. Supplementary Movie 12 shows the clockwise rotation of the C-Möbius[−1] actuator.

### Preparation of the B-Möbius[±*T*_w_] strip actuators

In general, a pre-crosslinked LCE796 strip was placed on the top of a pre-crosslinked LCE0 film, followed by heating in an oven at 60 °C for 2 days to spontaneously glue them together, to provide the bilayered LCE film which was then cut into a strip (38.0 mm long and 4.0 mm wide). Subsequently, one end of the straight bilayered LCE strip was twisted clockwisely either 180° or 360°, and further glued in a head-to-head manner to the other end by using a silicone adhesive (HJ-T326, Dongguan Fangguan Industrial Materials Co. Ltd.), to provide either B-Möbius[+1] or B-Möbius[+2] ribbon actuator. Similarly, an anti-clockwise twist of the bilayered LCE strip by 360° gave B-Möbius[−2] ribbon actuator instead.

### Preparation of the continuous rotating C-Möbius[±1] strip actuators

In general, the two asymmetric LCE pieces were overlapped with the thin and thick ends of the top piece stuck onto the thick and thin ends of the bottom piece, followed by heating in an oven at 60 °C for 2 days to spontaneously glue them together, to provide the bilayered LCE film which was then cut into a strip (33.0 mm long and 3.0 mm wide). Subsequently, one end of the straight bilayered LCE strip was twisted either clockwise or anticlockwise 180°, and further glued in a head-to-head manner to the other end by using a silicone adhesive (HJ-T326, Dongguan Fangguan Industrial Materials Co. Ltd.), to provide either C-Möbius[+1] or C-Möbius[−1] ribbon actuator.

## Supplementary information

Supplementary Information

Supplementary Movie 1

Supplementary Movie 2

Supplementary Movie 3

Supplementary Movie 4

Supplementary Movie 5

Supplementary Movie 6

Supplementary Movie 7

Supplementary Movie 8

Supplementary Movie 9

Supplementary Movie 10

Supplementary Movie 11

Supplementary Movie 12

Description of Additional Supplementary Files

## Data Availability

The source data underlying Figs. 3b, 4c, 5c, 6a–d, and 7b, e, g, and Supplementary Figs. 2, 7,e, f, and 13c are provided as a Source Data file with this paper. The data underlying all figures in the main text and Supplementary information are publicly available in the Figshare (DOI: 10.6084/m9.figshare.13614938) (ref. ^42^) at https://figshare.com/articles/dataset/Source_data/13614938. All other relevant data are available from the corresponding author on reasonable request. Source data are provided with this paper.

## Source data

Source Data
