# Supplementary material for: Light-driven continuous rotating Möbius strip actuators
Source: Nat Commun. 2021 Apr 20;12:2334. doi: 10.1038/s41467-021-22644-9 (PMC8058083; doi:10.1038/s41467-021-22644-9)
Supplement: Supplementary file 1 — Supplementary Information [file 41467_2021_22644_MOESM1_ESM.pdf]

Supplementary Information

Light-Driven Continuous Rotating Möbius Strip Actuators

Zhen-Zhou Nie[1,2,†], *Bo Zuo*[1,2,†], *Meng Wang*[1,2], *Shuai Huang*[1,2], *Xu-Man Chen*[1,2], *Zhi-Yang Liu*[1,2], *Hong Yang*[1,2]*

[1]Jiangsu Province Hi-Tech Key Laboratory for Bio-medical Research, State Key Laboratory of Bioelectronics, School of Chemistry and Chemical Engineering, Southeast University, Nanjing, 211189, China.

[2]Institute of Advanced Materials, Southeast University, Nanjing, 211189, China.

[†]Equal contributions.

*Correspondence and requests for materials should be addressed to H.Y. (email: yangh@seu.edu.cn).

Supplementary Figures

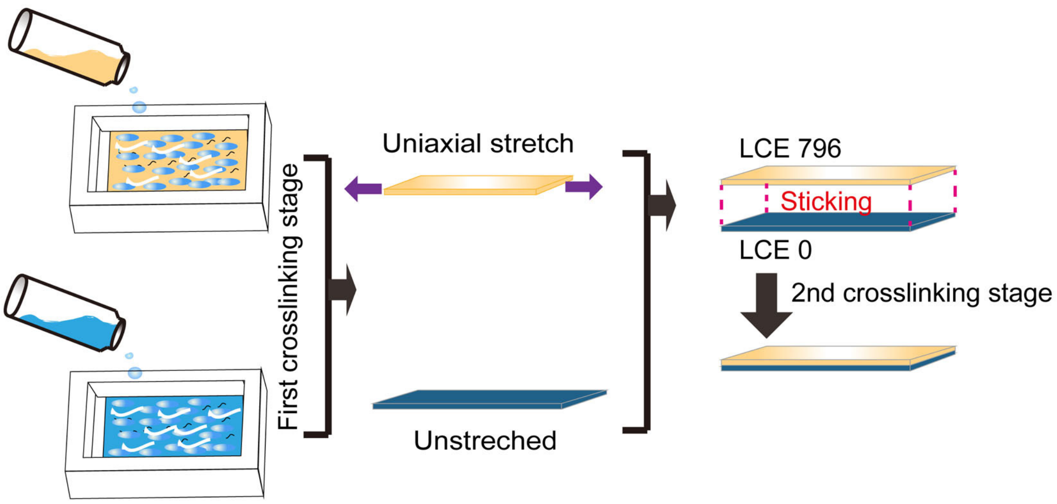

Supplementary Fig. 1 | Preparation procedures of bilayered LCE film. Schematic illustration of the preparation process of bilayered LCE film.

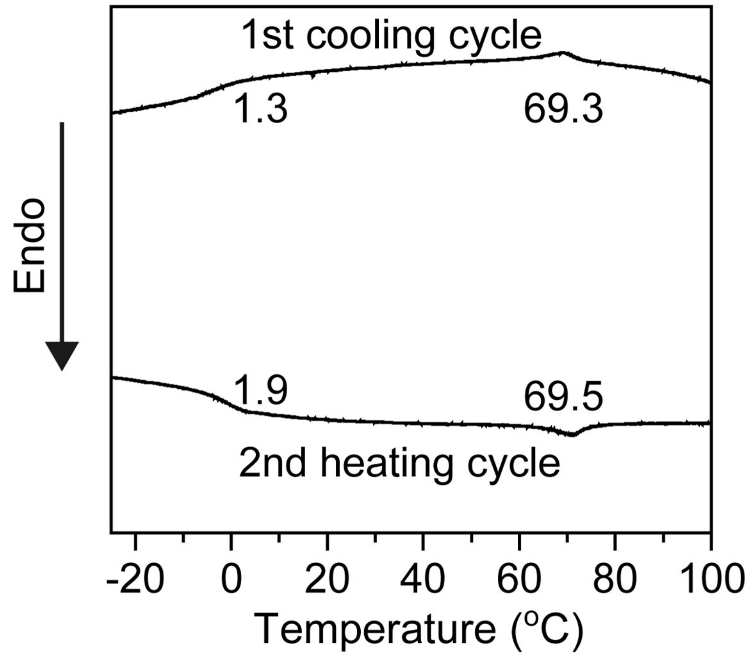

Supplementary Fig. 2 | DSC curves of LCE film. DSC curves of LCE796 during the first cooling and second heating scans at a rate of 10 ºC·min^{-1} under nitrogen atmosphere.

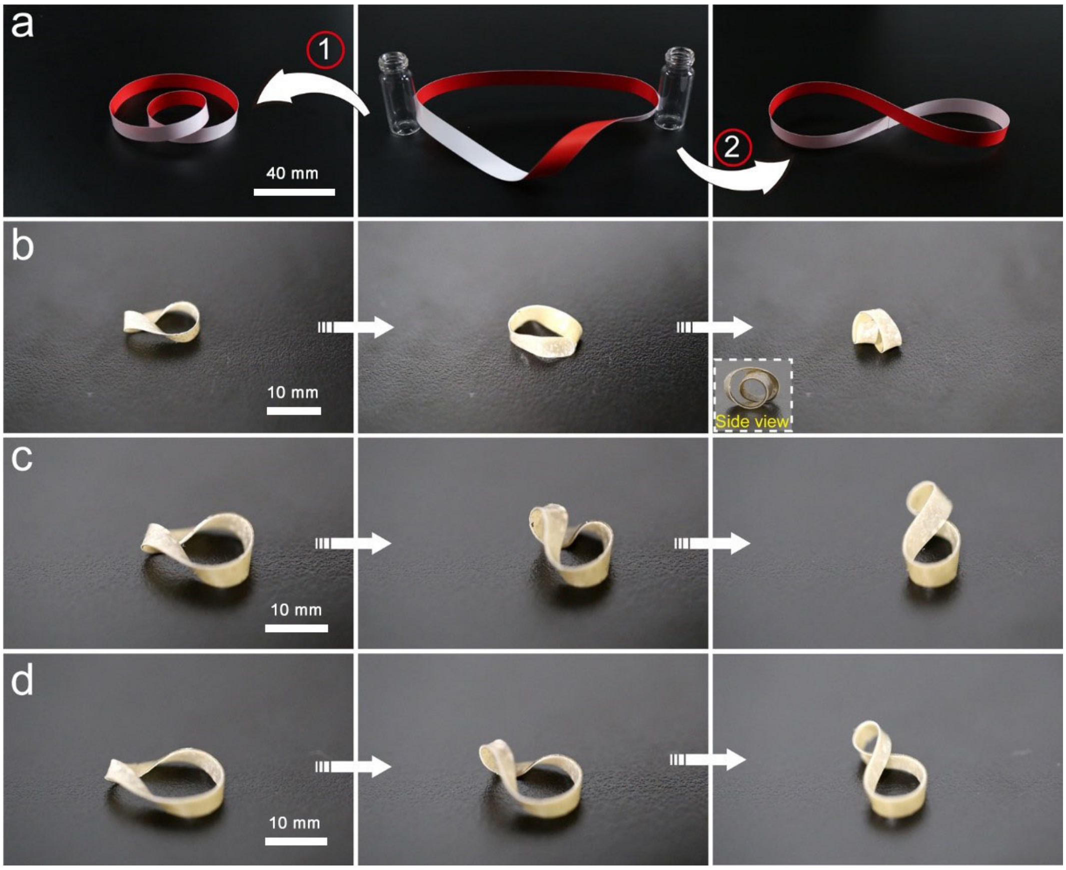

Supplementary Fig. 3 | Relationship between shape transformation and length/width ratios. **a** Demonstration of the transformation of one B-Möbius[+2] ribbon into either heart-shaped writhed loop or 8-figured writhed loop (scale bar: 40 mm). Photographs showing the shape deformations of three B-Möbius[+2] ribbon actuators with length/width ratios of **b** 10.5, **c** 11.8 and **d** 16.2 respectively, under the stimulation of NIR light irradiated on their locus of twist regions (scale bars: 10 mm).

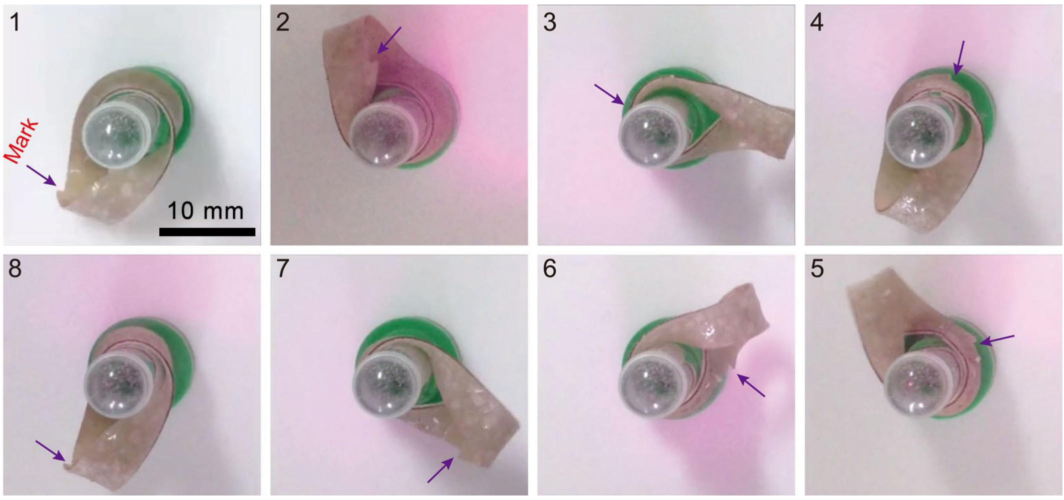

Supplementary Fig. 4 | Continuous clockwise rotation of B-Möbius[+2] actuator. An overlook view of the continuous clockwise rotation of a B-Möbius[+2] ribbon bearing a protrusion mark (scale bar: 10 mm). Supplementary Movie 4 shows the scenario in motion.

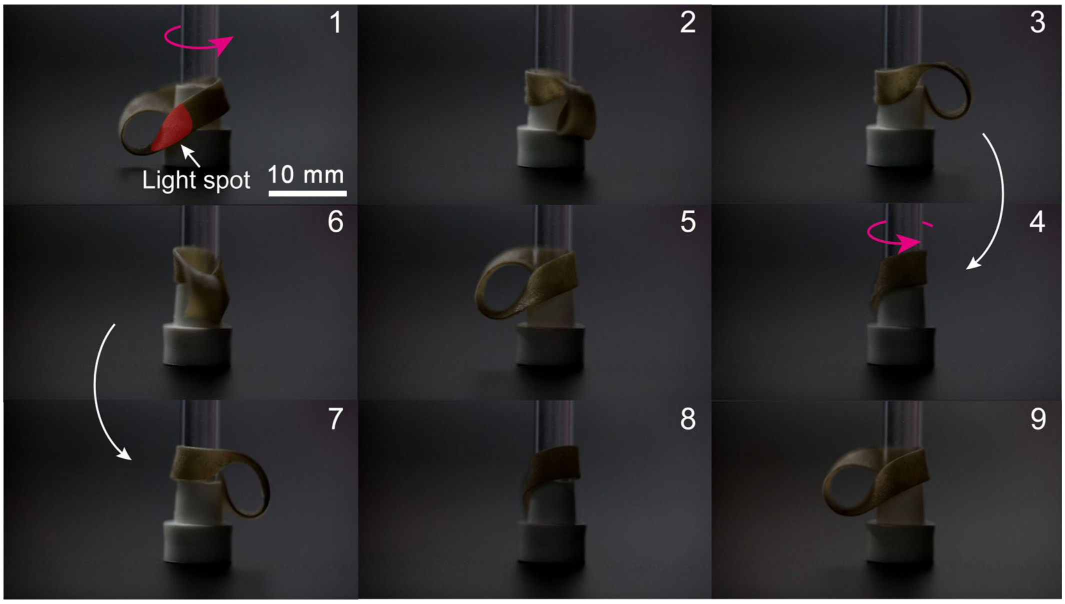

Supplementary Fig. 5 | Continuous anti-clockwise rotation of B-Möbius[-2] actuator around a circular cylinder. Photographs of continuous anti-clockwise rotation of one B-Möbius[-2] ribbon around a circular cylinder at a rotation rate of ca. 11 ° s^{-1} under the irradiation of NIR light (scale bar: 10 mm). Supplementary Movie 5 shows the scenario in motion.

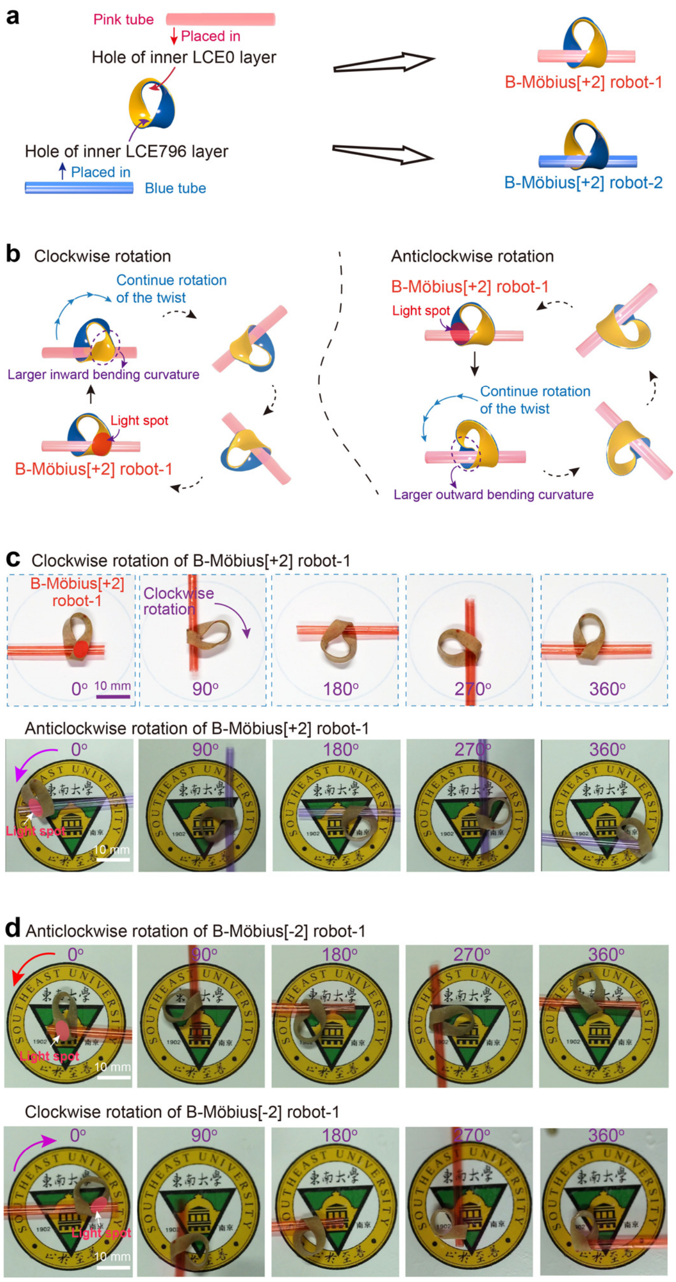

Supplementary Fig. 6 | Two types of B-Möbius[±2] rolling robots. a Schematic illustration of the fabrication of two types of B-Möbius[+2] robots (i.e., robot-1 and robot-2) where a plastic tube was placed in a Möbius strip hole with the inner layer composed of either LCE0 or LCE796. **b** Schematic illustration of the clockwise/anticlockwise rotation of B-Möbius[+2] robot-1 loaded with a pink cylindrical plastic tube (diameter: 3.0 mm, length: 38.0 mm). **c** Snapshots of clockwise (rotation rate: ca. 4.3 ° s^{-1}) and anticlockwise rotation (rotation rate: ca. 1.1 ° s^{-1}) of B-Möbius[+2] robot-1 (scale bars: 10 mm). Supplementary Movie 6 shows the scenario in motion. **d** Snapshots of anticlockwise (rotation rate: ca. 5.6 ° s^{-1}) and clockwise rotation (rotation rate: ca. 2.6 ° s^{-1}) of B-Möbius[-2] robot-1 (scale bars: 10 mm). Supplementary Movie 7 shows the scenario in motion.

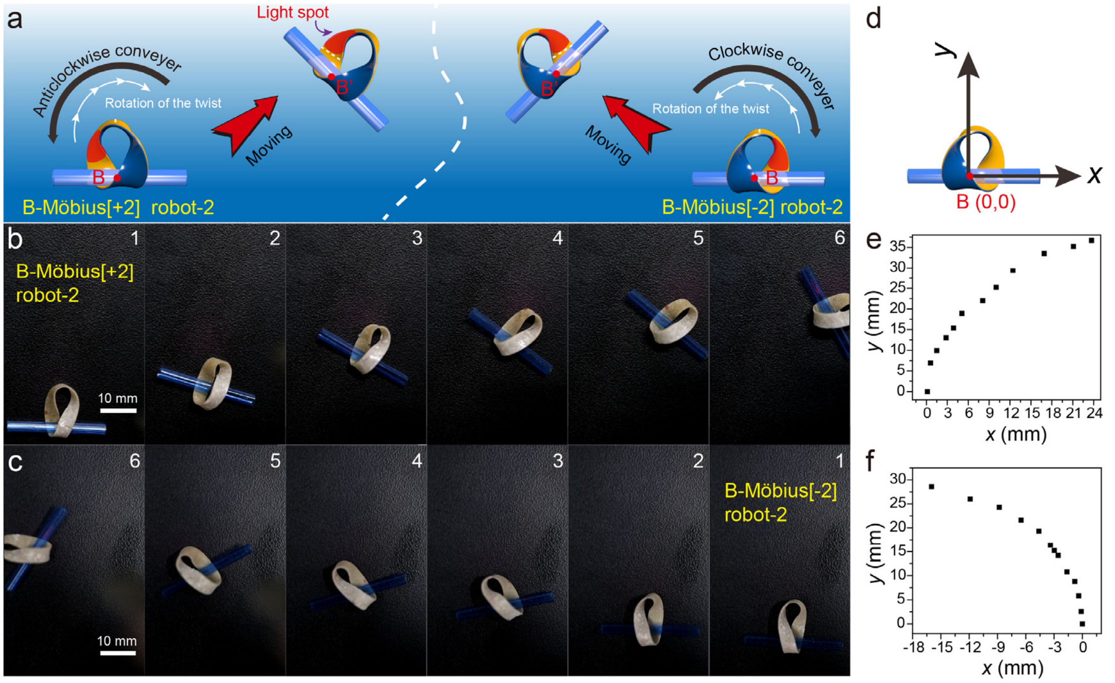

Supplementary Fig. 7 | Light-fueled B-Möbius[±2] rolling vehicle robot-2. **a** Diagrammatic drawing of the friction analysis and moving direction of B-Möbius[±2] robot-2 with a blue cylindrical plastic tube (diameter: 3.0 mm, length: 28.0 mm). Photographs of the rolling motions of **b** B-Möbius[+2] and **c** B-Möbius[-2] rolling robot-2 driven by NIR light (scale bars: 10 mm). Supplementary Movie 8 shows the scenario in motion. **d** Definition of the initial point in *x*-*y* coordinate system. Position coordinates of midpoint B of the hollow plastic tube recorded along the clockwise curvilinear motion path of **e** B-Möbius[+2] and **f** B-Möbius[-2] robot-2 respectively.

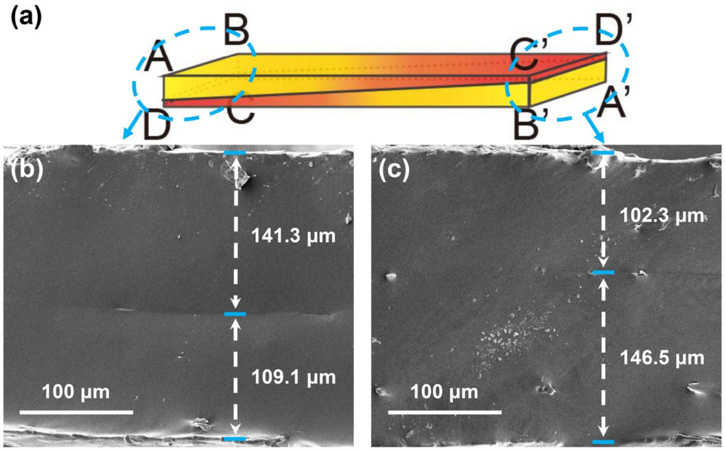

Supplementary Fig. 8 | SEM image of the cross-sectional area of the bilayered LCE film. SEM images showing the thickness of the two ends of a bilayered LCE film (scale bars: 100 μm).

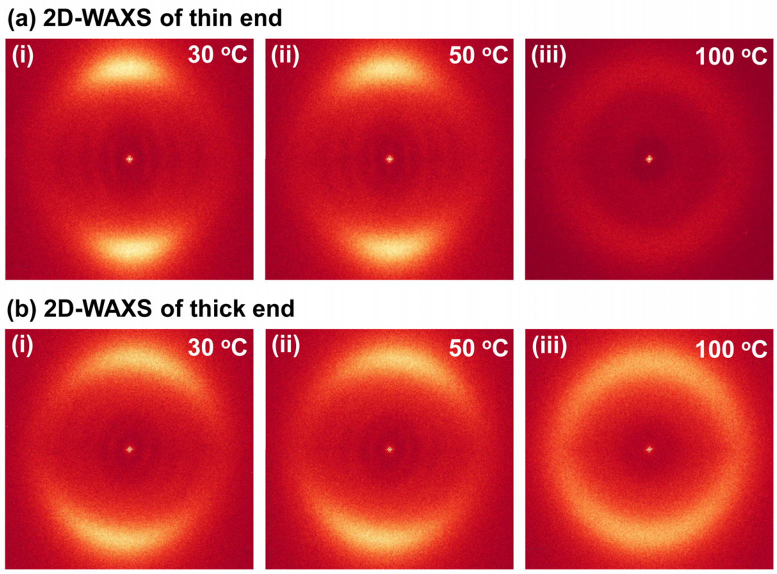

Supplementary Fig. 9 | Two-dimensional wide-angle X-ray diffraction patterns of the thin and thick end of LCE film. 2D WAXD patterns of **a** thin end and **b** thick end measured at 30 °C, 50 °C and 100 °C, respectively.

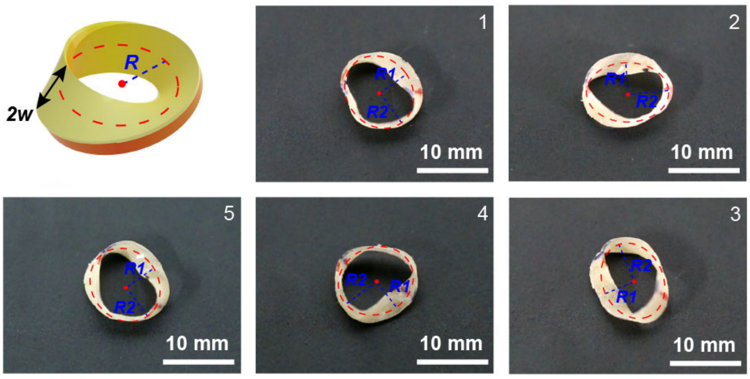

Supplementary Fig. 10 | Radius of the C-Möbius[+1] actuator. The radius (R = ca. 4.8 mm) of a C-Möbius[+1] actuator was measured by the averaged value of the radii in five Möbius actuator states in Supplementary Table 1. The width of the Möbius strip was $2w$ = ca. 3.0 mm, and the R/w ratio was ca. 3.2. Scale bars: 10 mm.

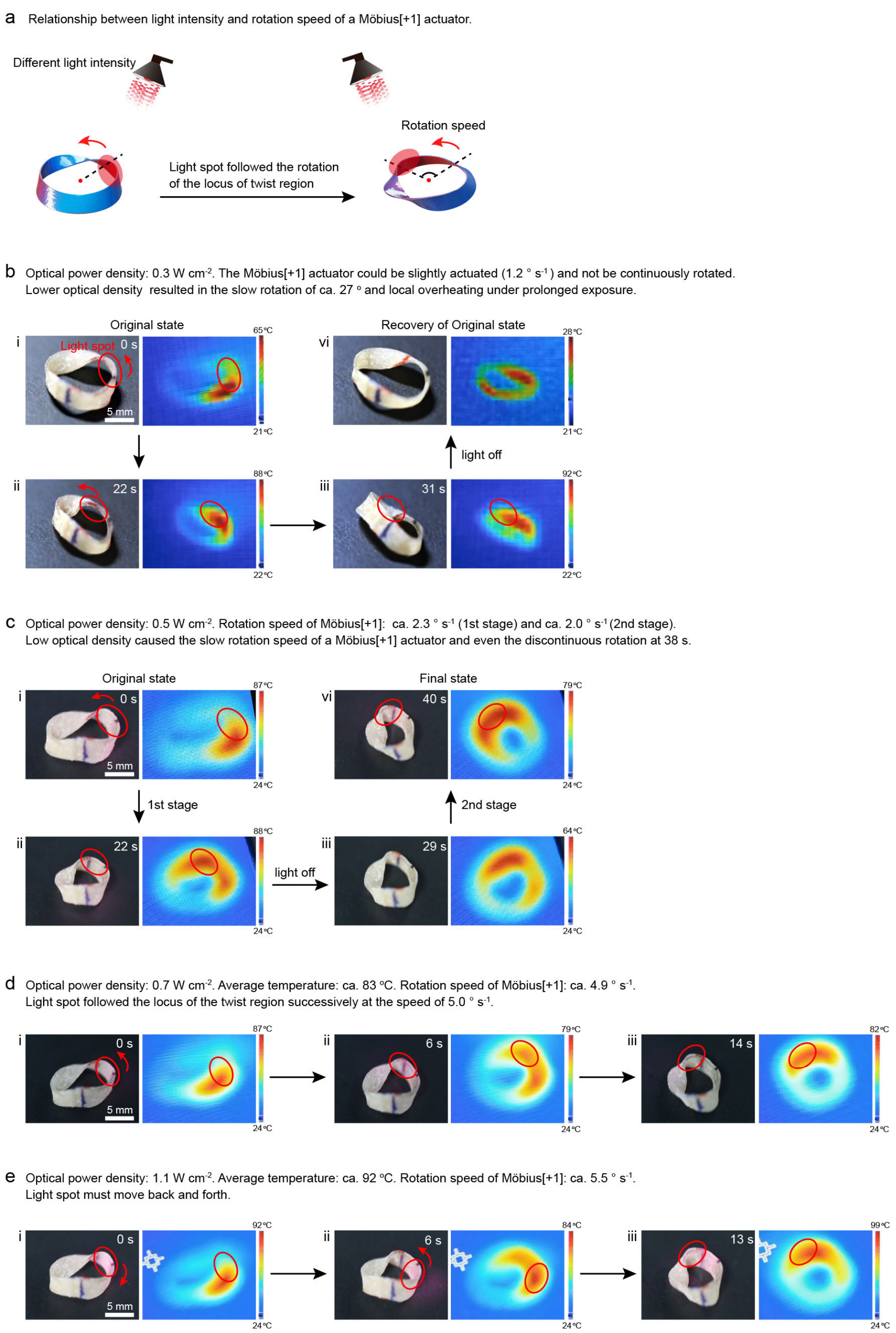

Supplementary Fig. 11 | Relationship between the optical intensity and the rotation speed of a C-Möbius[+1] actuator. a Schematic illustration of the rotation speed measurement at different optical intensities. The initial stimulus position near the locus of twist region was regarded as the start point of the rotation. The light spot followed the rotation trajectory of the locus of twist region. A rotation of ca. 70 degree was set as the test standard angle, in order to observe the rotation speed difference under varied light intensities: **b** 0.3 W cm^{-2}, **c** 0.5 W cm^{-2}, **d** 0.7 W cm^{-2}, and **e** 1.1 W cm^{-2} (scale bars: 5 mm).

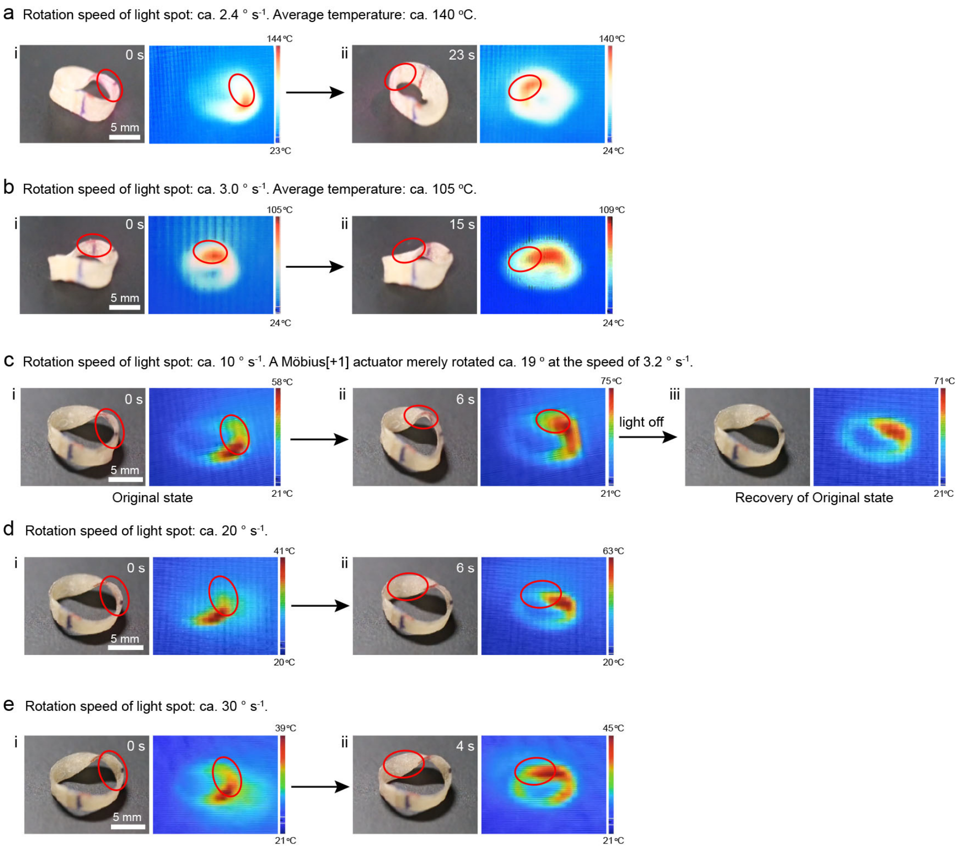

Supplementary Fig. 12 | Relationship between the light scanning rate and the rotation speed of a C-Möbius[+1] actuator. Photographs and thermal images of C-Möbius[+1] actuator rotating with a light scanning rate of **a** 2.4 ° s^{-1}, **b** 3.0 ° s^{-1}, **c** 10 ° s^{-1}, **d** 20 ° s^{-1}, and **e** 30 ° s^{-1}, respectively (scale bars: 5 mm).

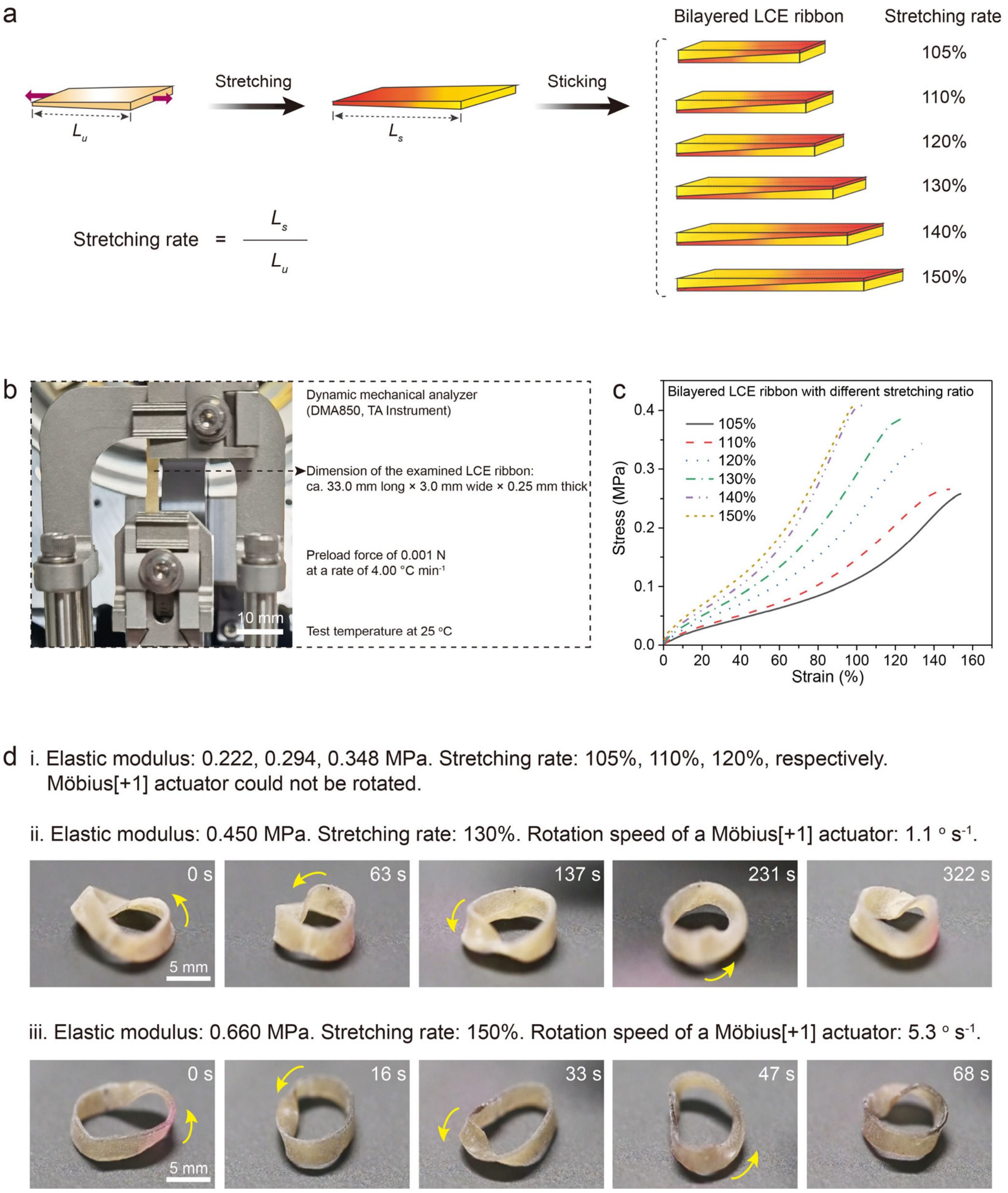

Supplementary Fig. 13 | Relationship between the elastic modulus and the rotation speed of a C-Möbius[+1] actuator. a Schematic illustration of the fabrication of the bilayered LCE ribbon with varied stretching rate. **b** Measurement conditions of dynamic mechanical analyzer with a tension clamp (scale bar: 10 mm). **c** Quasi-static stress-strain curves of bilayered LCE ribbons with different stretching rate measured at 25 °C. **d** Rotation capacity of the Möbius strip actuators with varied elastic modulus (scale bars: 5 mm).

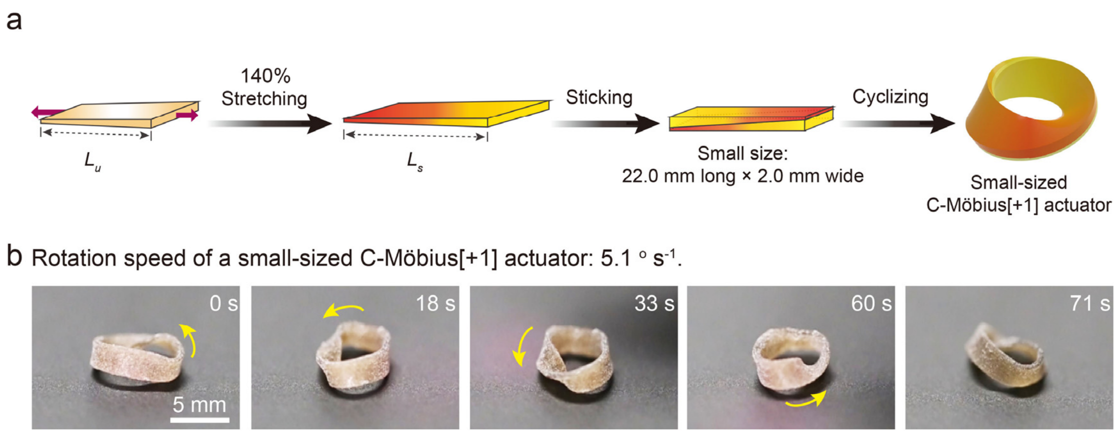

Supplementary Fig. 14 | Miniaturization of C-Möbius[+1] actuator. a Schematic illustration of the fabrication of a small-sized C-Möbius[+1] actuator (22.0 mm long and 2.0 mm wide). **b** Photographs of continuous anticlockwise rotation of the C-Möbius[+1] actuator at a speed of 5.1 ° s^{-1} (scale bar: 5 mm).

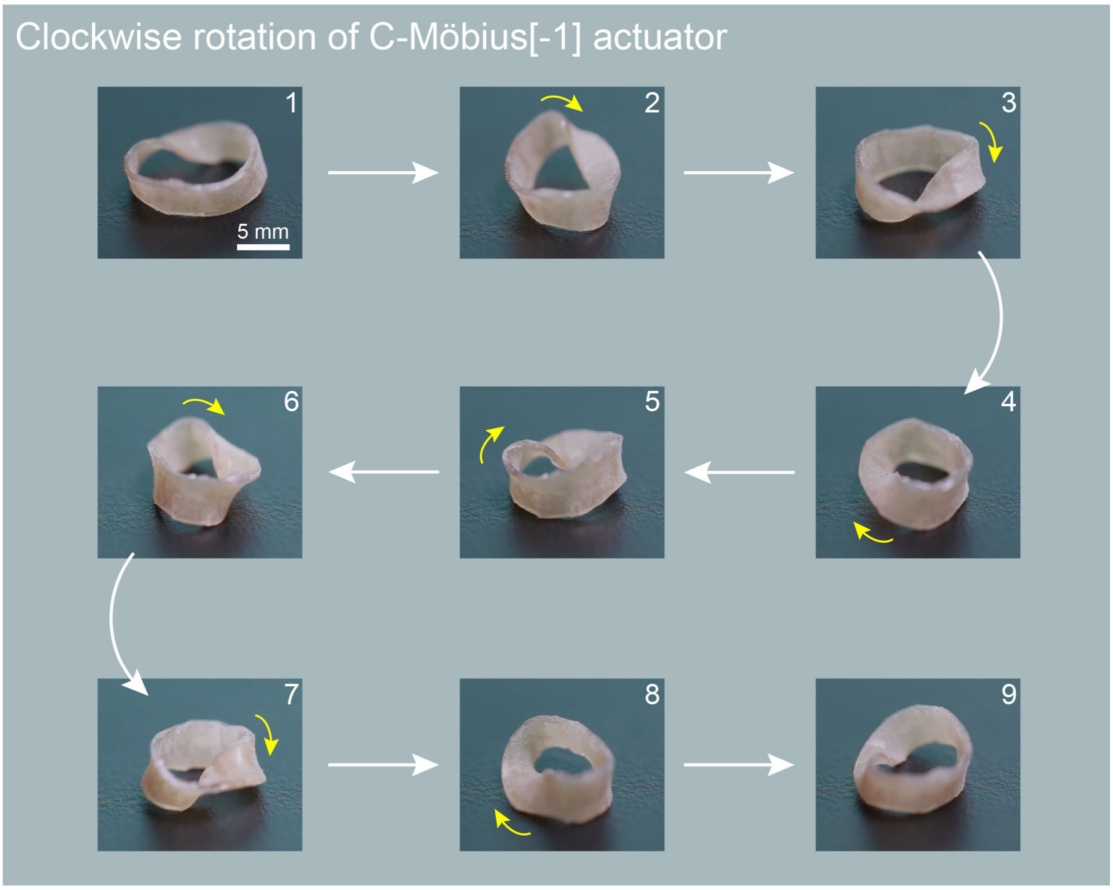

Supplementary Fig. 15 | Continuous clockwise rotation of C-Möbius[-1] strip actuator. Photographs of continuous clockwise rotation of the C-Möbius[-1] ribbon actuator at a speed of ca. 5.6 ° s^{-1} under the irradiation of NIR light (scale bar: 5 mm). Supplementary Movie 12 shows the scenario in motion.

Supplementary Tables

Supplementary Table 1 | Radius of the C-Möbius[+1] actuator measured in five different states.

Möbius actuator state	R1 (mm)	R2 (mm)	$R_{average}$[a] (mm)
1	4.5	5.0	4.8
2	4.6	5.0	4.8
3	4.6	4.9	4.8
4	4.7	4.9	4.8
5	4.7	4.8	4.8

[a]$R_{average}$ = (R1+R2)/2.

Supplementary Table 2 | Rotation speeds of Möbius actuators.

Rotation mode	Rotation speed[a] (degree s^{-1})	Light intensity[b] (W cm^{-2})	Supplementary Movie
Clockwise rotation of B-Möbius[+2] actuator around a circular cylinder	16	0.2	3
Anticlockwise rotation of B-Möbius[-2] actuator around a circular cylinder	11	0.2	5
Clockwise rotation of B-Möbius[+2] robot-1	4.3	0.2	6
Anticlockwise rotation of B-Möbius[+2] robot-1	1.1	0.2	6
Anticlockwise rotation of B-Möbius[-2] robot-1	5.6	0.2	7
Clockwise rotation of B-Möbius[-2] robot-1	2.6	0.2	7
Anticlockwise rotation of C-Möbius[+1] actuator	4.9	0.7	10
Clockwise rotation of C-Möbius[-1] actuator	5.6	0.7	12

[a]Rotation speed = Rotation angle/Rotation time. [b]Light intensity was measured by using a light power meter (Model: CEL-NP2000-2, Beijing Zhongjiao Jinyuan Technology Co., Ltd).

Supplementary Discussion

1. Relationship between the shape transformation and length/width ratio of B-Möbius[+2] ribbon actuator.

Upon irradiation with 808 nm NIR light of the locus of twist regions, one B-Möbius[+2] ribbon actuator with a length/width ratio of 10.5 (length: 42.0 mm, width: 4.0 mm) transformed into a heart-shaped loop, as shown in Supplementary Fig. 3b, whereas two Möbius[+2] ribbon actuators with length/width ratios of 11.8 (length: 47.0 mm, width: 4.0 mm) and 16.2 (length: 47.0 mm, width: 2.9 mm) became figure-eight loops, as presented in Supplementary Fig. 3c,d.

2. Two types of B-Möbius[±2] rolling robots.

B-Möbius[+2] robot-1 loaded with a pink tube (diameter: 3.0 mm, length: 38.0 mm) realized either clockwise or counterclockwise rotation under illumination on either the outside or inside ribbon region closely wrapped around the tube, whereas irradiation of any other regions of the ribbon by the light spot hardly made the ribbon move. As schematically demonstrated in Supplementary Fig. 6b, when the largely contractive LCE796 layer part of the twist region was illuminated by NIR light, the asymmetrical inward bending deformation contributed to an increase in the local curvature, and the Möbius ribbon underwent a rapid twisting transformation, which induced a friction thrust to drive successive clockwise rotation of B-Möbius[+2] robot-1 at a rotation rate of ca. 4.3 ° s^{-1}. To reverse the rotational direction of B-Möbius[+2] robot-1, under light illumination of the LCE0 layer part of the twist region around the tube, outward bending deformation was realized that induced a change in the local curvature and further generated a continuous friction thrust in the opposite direction, which brought about an anticlockwise rotation of B-Möbius[+2] robot-1 at a speed of ca. 1.1 ° s^{-1}, as illustrated in Supplementary Fig. 6b,c and Supplementary Movie 6. Thus, the specific light-stimulus location was critical for the bidirectional rotation of a single rolling robot. Moreover, the rotation motions of B-Möbius[-2] robot-1 were also examined, as demonstrated in Supplementary Fig. 6d and Supplementary Movie 7.

B-Möbius[±2] robot-2 loaded with a blue hollow plastic tube (diameter: 3.0 mm, length: 28.0 mm) placed in contact with the inner LCE796 layer could move along a curvilinear trajectory under light stimulation of a position near the twist region of the B-Möbius[±2] actuator. Likewise, B-Möbius[±2] robot-2 remained unchanged upon light spot irradiation of any other region of the strip. As shown in Supplementary Fig. 7a, when the NIR light stimulated a position near the locus of twist region of the actuator, the ribbon twist tended to rotate clockwise/anticlockwise, which was however hampered by the table and then had to stop; the generated internal stress induced a reverse thrust that forced the ribbon to frictionally slide on the table, behaving like an anticlockwise/clockwise conveyer belt.

To track the movement paths of B-Möbius[±2] rolling robot-2, the real-time

position coordinates of one midpoint, B, of the hollow plastic tube were also analysed by Tracker software (Supplementary Fig. 7d). Under the action of reverse thrust opposite to the twist rotation direction, B-Möbius[+2] robot-2 rolled ca. 45 mm within 66 s along with a clockwise curvilinear trajectory, whereas B-Möbius[-2] robot-2 rolled ca. 34 mm within 45 s in an anticlockwise curvilinear trajectory, as illustrated in Supplementary Fig. 7e,f and Supplementary Movie 8. This experiment demonstrated that these B-Möbius[±2] actuators had potential applications in conveyer and motor devices, etc.

3. Boltzmann function for data fitting.

Boltzmann function was used for fitting the data to obtain a sigmoidal curve.

Boltzmann equations are presented as below:

$$\beta = \frac{A_1 - A_2}{1 + e^{(\alpha - \alpha_0)/k}} + A_2$$

$$A_1 = 0.71981 \pm 3.56716$$

$$A_2 = 172.91154 \pm 2.56053$$

$$\alpha_0 = 80.21075 \pm 1.12744$$

$$k = 17.00084 \pm 1.28714$$

where A_1 is the initial flip angle, A_2 is the final flip angle, α_0 is the twist locus rotation angle required to reach the half flip angle value from A_1 to A_2, and k is the constant.

Supplementary Methods

General Considerations. Polymethylhydrosiloxanes (HMS-993, M.W. 2200-2400) were purchased from Gelest Inc. Toluene was redistilled from sodium and sodium benzophenone ketyl under nitrogen. The mesogenic monomer 4-methoxyphenyl-4-(1-buteneoxy)benzoate (MBB) and the crosslinker 1,4-bis-undec-10-enyloxy-benzene (11UB), 4-[4-(vinyloxy)butoxy]phenyl 4-[4-(vinyloxy)-butoxy]benzoate (VBPB) were synthesized following literature protocols[1-3].

Optical images and movies were recorded by using a Canon EOS60D camera. Differential scanning calorimetry (DSC) spectra were measured on a TA Q100 instrument (New Castle, DE) under nitrogen purge at a heating/cooling rate of 10 °C·min^{-1}. A MW-GX-808 NIR instrument (Changchun Laser Optoelectronics Technology Co., Ltd., China) was used as NIR light stimulus source.

Preparation of pre-crosslinked LCE796 film.

PMHS (50.0 mg, 0.83 mmol Si-H groups), MBB (198.7 mg, 0.67 mmol), 11UB (34.5 mg, 0.083 mmol) were dissolved in 2.5 mL toluene. Then, the mixture was transferred to a polytetrafluoroethylene (PTFE) rectangular mould (4.0 cm long, 2.0 cm wide and 1.5 cm deep). Subsequently, 50 μL CH_2Cl_2 solution containing 0.2 mg YHD796 and 5.0 μL Karstedt catalyst solution (platinum(0)-1,3-divinyl-1,1,3,3-tetramethyldisiloxane complex solution in xylene, Pt ~2%) were added into the above mould, followed by an ultrasonication process for 1.5 min. After heating in an oven at 60 ºC for 3 h, the pre-crosslinked LCE796 film was carefully taken out, cut into a strip and further uniaxially stretched to ca. 140 % of its original length (4.0 cm long and 1.0 cm wide).

Preparation of pre-crosslinked asymmetric LCE796 film.

PMHS (50.0 mg, 0.83 mmol Si-H groups), MBB (198.7 mg, 0.67 mmol), 11UB (34.5 mg, 0.083 mmol) were dissolved in 2.5 mL toluene. Then, the mixture was

transferred to a polytetrafluoroethylene (PTFE) rectangular mould (4.0 cm long, 2.0 cm wide and 1.5 cm deep). Subsequently, 50 μL CH_2Cl_2 solution containing 0.2 mg YHD796 and 5.0 μL Karstedt catalyst solution (platinum(0)-1,3-divinyl-1,1,3,3-tetramethyldisiloxane complex solution in xylene, Pt ~2%) were added into the above mould, followed by an ultrasonication process for 1.5 min. Then, the PTFE mould was placed in an inclined surface with a tilt angle of 10° and heating in an oven at 60 °C for 3 h. The pre-crosslinked LCE796 film was carefully taken out, uniaxially stretched to ca. 140 % of its original length (4.0 cm long and 1.0 cm wide) and further cut into two symmetric pieces.

Preparation of pre-crosslinked LCE0 film.

PMHS (50.0 mg, 0.83 mmol Si-H groups), MBB (198.7 mg, 0.67 mmol), 11UB (6.9 mg, 0.017 mmol), VBPB (28.3 mg, 0.067 mmol) and 5.0 μL Karstedt catalyst solution (platinum(0)-1,3-divinyl-1,1,3,3-tetramethyldisiloxane complex solution in xylene, Pt ~2%) were dissolved in 2.5 mL toluene, following by ultrasonication for 1.5 min in a PTFE rectangular mould (6.0 cm long, 2.0 cm wide and 1.5 cm deep). After heating in an oven at 60 °C for 2 h, the pre-crosslinked LCE0 film was carefully taken out and cut into a strip (4.0 cm long and 1.0 cm wide) without any mechanical stretching treatment.

Supplementary References

1. Li, C., Liu, Y., Huang, X. & Jiang, H. Direct sun-driven artificial heliotropism for solar energy harvesting based on a photo-thermomechanical liquid-crystal elastomer nanocomposite. *Adv. Funct. Mater.* **22**, 5166-5174 (2012).

2. Li, C., Liu, Y., Lo, C. W. & Jiang, H. Reversible white-light actuation of carbon nanotube incorporated liquid crystalline elastomer nanocomposites. *Soft Matter* **7**, 7511-7516 (2011).

3. Sanchez-Ferrer, A. & Finkelmann, H. Uniaxial and shear deformations in Smectic-C main-chain liquid-crystalline elastomers. *Macromolecules* **41**, 970-980 (2008).
