# Supplementary material for: Light-driven continuous rotating Möbius strip actuators
Source: Nat Commun. 2021 Apr 20;12:2334. doi: 10.1038/s41467-021-22644-9 (PMC8058083; doi:10.1038/s41467-021-22644-9)
Supplement: Supplementary file 14 — Description of Additional Supplementary Files [file 41467_2021_22644_MOESM14_ESM.pdf]

Title: Supplementary Movie 1
Description: presents the actuation behavior of S-Möbius[+1] and S-Möbius[+2] strips.

Title: Supplementary Movie 2
Description: shows the actuation behavior of B-Möbius[+1] and B-Möbius[+2] strips under the stimulation of NIR light.

Title: Supplementary Movie 3
Description: shows the clockwise rotation of B-Möbius[+2] actuator around a circular cylinder.

Title: Supplementary Movie 4
Description: presents a top view of the clockwise rotation of B-Möbius[+2] actuator around a circular cylinder.

Title: Supplementary Movie 5
Description: shows the anticlockwise rotation of B-Möbius[-2] actuator around a circular cylinder.

Title: Supplementary Movie 6
Description: demonstrates the clockwise and anticlockwise rotation of B-Möbius[+2] robot-1 loaded with a cylindrical tube.

Title: Supplementary Movie 7
Description: presents the anticlockwise and clockwise rotation of B-Möbius[-2] robot-1 loaded with a cylindrical tube.

Title: Supplementary Movie 8
Description: shows the rolling motions of B-Möbius[+2] robot-2 and B-Möbius[-2] robot-2 driven by NIR light.

Title: Supplementary Movie 9
Description: shows a light-fueled B-Möbius[+2] tractor dragging a foamy cylinder.

Title: Supplementary Movie 10
Description: shows the anticlockwise rotation of C-Möbius[+1] actuator.

Title: Supplementary Movie 11
Description: indicates a side view of the anticlockwise flip motion of C-Möbius[+1] actuator.

Title: Supplementary Movie 12
Description: shows the clockwise rotation of the C-Möbius[-1] actuator.
